# Methods of Analysis of the Nasal Profile: A Systematic Review with Meta-analysis

**DOI:** 10.1155/2021/6680175

**Published:** 2021-03-15

**Authors:** Agnieszka Jankowska, Joanna Janiszewska-Olszowska, Maciej Jedliński, Katarzyna Grocholewicz

**Affiliations:** ^1^Private Practice “Dental Clinic Jankowscy” Zary, Poland; ^2^Department of Interdisciplinary Dentistry, Pomeranian Medical University in Szczecin, Poland

## Abstract

The nose is the most prominent structure of the face, influencing facial appearance and profile. Orthodontists have an awareness of facial structures, including nasal morphology, when diagnosing and treatment planning. Maxillofacial surgeons influence facial profile by bimaxillary surgery, improving facial aesthetics and harmony. The aim of this review was to summarize the available methods of analysing nasal morphology and profile, and to assess their complexity. A literature search was conducted in PubMed, Scopus, Web of Science, and Embase using the following search terms: “nasal profile analysis”, “nasolabial angle”, and “nasal profile cephalometric” in order to select studies providing knowledge on correlations between occlusion and nasal development, differences between skeletal classes, ethnic variability, and differences between the sexes. Studies concerning genetic disorders were excluded. Finally, 17 full-text papers were analysed, which pertained to nasolabial angle, or facial profile including the nose. Data concerning methods, ethnic group, reference landmarks used, and measurements made were extracted and placed in tables. Numerous methods of nasal profile analysis can be found in the literature. These methods describe various numbers of parameters, which have influence on facial aesthetic. Nasal parameters are correlated to skeletal class and nasolabial angle, positions of upper incisors, and maxillary inclination.

## 1. Background

The nose is the most prominent element of the face, influencing facial appearance and profile [1–5]. According to the study by Ghorbanyjavadpour and Rakhshan [6], there are some factors associated with the esthetics of the soft-tissue profile, also associated with the nose, such as less prominent noses with higher tips and subnasales anterior to the upper lip. Numerous authors deal with analysis of the facial profile [7–15]. The studies take into consideration age, sex, skeletal class, and ethnic group. Maxillofacial surgeons influence facial profile by bimaxillary surgery, improving facial aesthetics and harmony [16], as well as nasal projection and nasolabial angle (NLA) [17]. Nasal growth since early childhood as well as nasal shape and profile has been subjected to various analyses by numerous authors [1–3, 7, 9, 14, 18–22]. Orthodontists have an awareness of facial structures, including nasal morphology, when diagnosing and treatment planning in order to achieve good results after treatment cessation [11]. The aim of this review was to summarize the available methods of analysing nasal morphology and profile and to assess their complexity.

## 2. Material and Methods

### 2.1. Search Strategy

The search and the entire review were performed according to the PRISMA statement [23] and following the guidelines from the Cochrane Handbook for Systematic Reviews of Interventions [24]. All searching was performed using a combination of different MeSH terms and free-text terms. After all, the final search strategy was determined by several presearches. The literature search was conducted in following databases: PubMed, Scopus, Web of Science, and Embase using the following search terms: “nasal profile analysis” OR “nasolabial angle” OR “nasal profile cephalometry” on 6^th^ December 2020. The papers initially selected were subjected to detailed analysis, regarding the methods of analysis as well as knowledge on nasal morphology and development.

### 2.2. Eligibility Criteria

The following inclusion criteria were employed for this review: (1) randomized clinical trials (RCTs); (2) analytical studies; (3) observational studies; (4) studies on human, healthy subjects; (5) studies published in English.

Then, the following exclusion criteria were employed for this review: (1) case reports; (2) reviews; (3) abstract and author debates or editorials; (4) lack of effective statistical analysis; (5) studies concerning congenital deformities; (6) studies evaluating theoretical algorithms, classification systems, or descriptions of protocols. No limitation referring to the year of publication of the studies was imposed.

All papers found were analysed in order to select studies providing knowledge on correlations between occlusion and nasal development, differences between skeletal classes, ethnic variability, and differences between the sexes.

### 2.3. Data Extraction

Titles and abstracts were selected independently by two authors (MJ and AJ), following the inclusion criteria. The full text of each identified primarily included article was then analysed to find out whether it was appropriate for inclusion. Disagreements were resolved through discussion with the team supervisor (JJO). Authorship, year of publication, data concerning methods, ethnic group, reference landmarks used, and measurements taken were independently extracted by two authors (AJ and MJ) and examined by the third author (JJO).

### 2.4. Risk of Bias

According to the PRISMA statements, the evaluation of methodological quality gives an indication of the strength of evidence provided by the study because methodological flaws can result in bias [23]. For studies based on the observation of structures found in radiological examinations, a specific scale for Clinical Studies of Radiologic Examinations should be applied. For this reason, it was decided to use the Arrive´ Scale [25]. It consists of 15 components, i.e., study design, study purpose, reference standard, inclusion criteria, indeterminate results, exclusion criteria, spectrum of patients, analysis method, analysis criteria, avoided work-up bias, avoided diagnostic-review bias, avoided test-review bias, intraobserver reliability, interobserver reliability, and statistical analysis, that accurately assess the bias risk, and due to their complexity, they provide detailed analysis of the results. One point is given for the compliance of the test characteristics with the required characteristics listed in the scale. In the event of a defect in the methodology, the research receives 0 points. The more points the research received, the better the methodology it has.

### 2.5. Meta-analysis

Meta-analysis was performed using random-effects model via metafor and compute.es R packages [26] with Standardized Mean Differences (SMD) and 95% confidence intervals (95% CI) being calculated as effect estimates. Heterogeneity was assessed quantitatively using *I*^2^-statistics and Cochran's *Q*. [27]. The meta-analysis included studies that examined the values of nasiolabial angle separately for women and men and provided SD values for both groups.

## 3. Results

**Figure 1:**
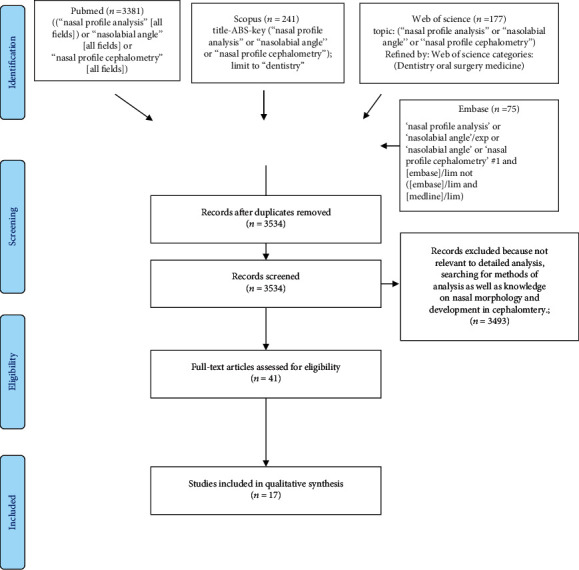
PRISMA 2009 flow diagram.

The search strategy identified 3874 potential articles: 3381 from PubMed, 241 from Scopus, 177 from Web of Science, and 75 from Embase. After duplicates had been removed, 3534 articles were screened. After that, 3493 papers were excluded because they did not correspond with the topic of this review. Of the remaining 41 papers, 24 were excluded because they were not relevant to the eligibility criteria. Finally, 17 full-text papers were included into qualitative analysis (Figure 1 PRISMA 2009 Flow Diagram). All of included studies pertained to nasolabial angle, or facial profile, including the nose.

The study material of the studies included is presented in Table 1.  Table 2 presents nasal and cephalometric landmarks from the literature. Angular and linear variables from the studies included have been described in Table 3.

### 3.1. Risk of Bias

The Arrive Scale was chosen in order to unify quality assessment of all studies included in this systematic review. If a decision was made to choose less specific, more popular scales, such as Newcastle-Ottawa scale or Jadad scale, it would cause chaos due to overdivision through various types of research, so the results of the risk of bias assessment would not be transparent. (Table 4).

### 3.2. Meta-analysis

Many of the studies included in the review leave the question open as to whether gender influences the nasiolabial angle. It was concluded that it is worth performing metanalysis in order to unify the results included in the review of studies and draw a common, consistent conclusion. There were 8 included studies in metanalysis. The values and SD of NLA that were reported are presented in Table 5.

**Figure 2:**
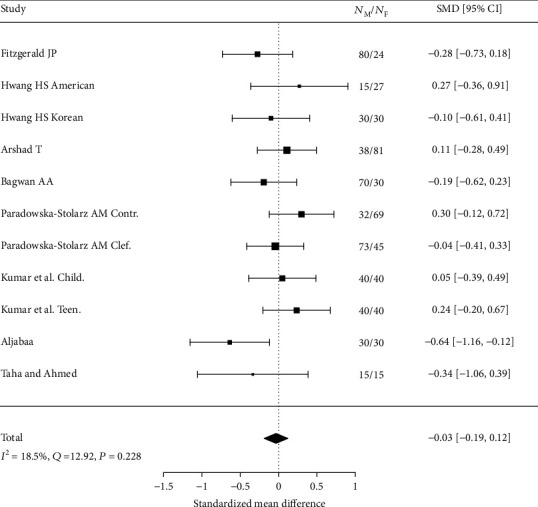
Forest plot of 8 studies on gender influence on the value of nasiolabial angle.

The results by Hwang et al. [28], Paradowska–Stolarz and Kawala [29], and Kumar et al. [30] are presented separately (in 2 separate groups) because of the significant factors differing study groups. The results are shown in Figure 2. SMD should be treated as measure of gender influence on the value of nasiolabial angle. Positive value of SMD indicates greater angle in male patients, negative—in female patients.

**Figure 3:**
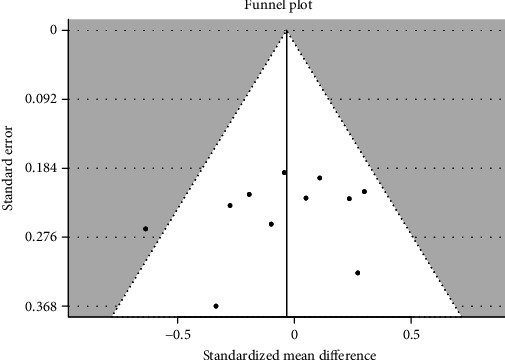
Funnel plot of 8 studies does not reveal publication bias.

Forest plot of 11 studies on gender influence on the value of nasiolabial angle has been presented in Figure 2. Positive value of SMD indicates a higher angle in male patients, negative—in female patients. Gender has an insignificant (*p* = 0.671) negative effect size. Study results are consistent—heterogeneity is insignificant (*p* = 0.228); only 18.5% of the variability come from heterogeneity. Funnel plot (Figure 3) does not reveal publication bias.

## 4. Discussion

Part of the methodology that is missing in many of the studies included in the review is patient selection and comparability. The number of women and the number of men were in some studies unequal [29, 31–33] This deficiency was often caused by the randomization procedure (selection of cephalograms); sometimes, it was a simple negligence of researchers. An important thing, which was also missing in many papers, was the analysis of cephalograms by more than one observer [28, 29, 31, 32, 34–37]. Even if intraobserver reliability is ensured by performing more than one cephalometric analysis of each radiograph, it is important to have another researcher or even computer AI as verification of identification of cephalometric landmarks. This is noticeable that more recent research places more emphasis on this aspect of the methodology. Due to the common standardization of cephalometric analysis and the additional description of the position of the points in every paper, there were no objections in such parts of evaluation as reference standard, indeterminate results, or in the method and criteria of the analysis. These attributes result from the subject of research (the principles of cephalometric analysis are well known and well-established), not from the way it was conducted.

The present study is a summary of the available methods of analyzing nasal profile on lateral cephalograms. All landmarks and measurements have been tabularized. Moreover, scientific findings on correlations between nasal and craniofacial morphology as well as age, gender, and ethnicity have been summarized. The results of this systematic review may aid treatment planning, when facial and esthetics are the primary goal.

In 2006, Gulsen et al. [38] published an analysis of the nasal profile, which aimed to find a correlation between craniofacial and nasal morphology. The authors of the study cited analysed 12 nasal variables in 262 patients aged 18-30 with no history of surgical or orthodontic treatment. Tables 2 and 3 present reference landmarks as well as linear and angular measurements of the nasal structures analysed in the vertical and sagittal planes. Based on these results, Gulsen et al. [38] defined numerous nasal parameters describing shape, size, the presence of dorsal curvatures, and nasal length or depth. The same method of nasal analysis was later used by Arshad et al. [31] on cephalometric radiographs of 119 subjects aged 18-40, with no congenital deformities and no history of orthodontic treatment, in order to assess nasal profile in the sagittal and vertical planes and analyse sexual dimorphism. The variables measured on cephalometric radiographs are presented in Table 3.

Nehra and Sharma [34] analysed correlation between vertical skeletal pattern of the maxilla and nasal morphology on 190 pretreatment lateral cephalometric radiographs of patients aged 18-27 years. The cephalometric variables used can be found in Table 3. They found a correlation between nasal parameters (length, depth, tip angle, and nasolabial angle), maxillary and mandibular inclinations, and anterior and posterior facial heights. They found significant correlations between nasal values (length and nose tip angle) and position of the maxilla.

Changes in nasal growth, size, and morphology referring to the vertical pterygomaxillary plane (PMV) were described by Meng et al. [39] based on 305 cephalometric radiographs of 23 females and 17 males aged 7-18 years. The reference landmarks, lines, and angles are presented in Tables 2 and 3. Between the ages of 7-18 years, an increase of both the upper and lower nasal height was found. However, at the age of 7, upper nasal height achieved 80% of its final measure. Moreover, girls had 90% of their final nasal height already at the age of 7, boys after the age of 17. Nasal depth was 70% of its final measure in girls at the age of 7, in boys at the age of 11. Nasal measurements were always lower in girls than in boys.

A single study by Buschang et al. [40] was found describing a growth analysis of the upper and lower parts of the nasal dorsum in children and adolescents. The nasal dorsum appeared to grow on average by 10° between 6 and 14 years of age. Its development is correlated with nasal tip growth. In subjects with a horizontal skeletal growth pattern, the dorsum moves upwards and forwards. In the case of vertical growth, rotations occur directed downwards and backwards.

The correlation between skeletal patterns and nasal shape was analysed by Robinson et al. [37] in 123 women, based on two angles and three linear measurements (Table 3). A straight nasal dorsum was more prevalent in skeletal class I, convex (nasal hump)—in class II, whereas concave—in class III. Nasal length and depth were strongly correlated with age.

Skinazi et al. [35] focused on analyzing nasal surface area in a French population. They drew Rickett's Esthetic Line (from nasal tip to the most prominent chin point) and Juanita line (drawn from Sn (the depth of the nasolabial sulcus) to the deepest point of the labiomental sulcus) on facial profile and calculated surface areas of the nose, lips, and chin. Mean nasal surface area was 246.14 ± 65.51 mm^2^ in women and 235.4 ± 59.16 mm^2^ in men.

Eight papers were found pertaining to NLA. The authors of the studies cited [28, 31–33] analysed variability in nasal morphology. The data extracted are presented in Table 5.

Both Arshad et al. [31] and Gulsen et al. [38] found a significant correlation between nasomental angle (NMA) and convexity of facial soft tissue (SFC) and skeletal class. NMA values are higher in skeletal class III, lower in class II [31, 38]. A higher SFC angle is found in class II, a lower in class III [31, 38]. Moreover, Gulsen et al. [38] found correlation between NLA and skeletal class. Arshad et al. [31] indicated that concavity of the lower part of the nasal dorsum (Dconv) is strongly dependent on skeletal class. Numerous authors proved significant sexual dimorphism concerning individual nasal variables. The results indicate that both nasal length [31, 37–39, 41, 42] and nasal depths (Ndepth1, Ndepth2) [31, 37, 38] have higher values in men than in women. Men are also characterized by higher values of the variable describing nasal dorsum convexity (Hump) [31, 38]. On the other hand, no significant correlations were found between skeletal class and the size of the nasal dorsum hump [31, 38]. Gulsen et al. [38] stated that NLA in class II is higher than in classes I and III.

Numerous authors [7–10, 28, 29, 31–34, 38, 43–47] have used NLA, enabling them to assess nasal position in the facial profile and, indirectly, the position of maxillary anterior teeth. NLA is very important for orthodontic treatment planning and is easy to measure. Table 5 shows that the values of NLA differ between ethnic groups: they are lowest in Korean and higher in European-American adults. NLA is similar in both sexes.

The following correlations were found between nasal structures and facial skeleton [38]: Ndepth1—positive correlation with mandibular length, posterior facial height, and hump; Nlength—positive correlation with anterior and posterior face height, maxillary and mandibular length, hump, and nasal bone length; Ndepth2—positive correlation with maxillary length and columella convexity; Hump—positive correlation with anterior and posterior face height, columella convexity, and nasal bone length; NLA—positive correlation with SFC, NBA; NMA—weak positive correlation with mandibular length and position; SFC—weak positive correlation with maxillary position, mandibular inclination, and strong positive correlation with facial convexity.

Arshad et al. [31] reported the following findings: skeletal classes I, II, and III are characterized by different nasal profiles due to different values of NLA, soft tissue convexity, and low convexity of the nasal dorsum and significant differences exist between women and men concerning nasal profiles in terms of nasal length, nasal depth, hump, convexity columella, and nasal bone length.

In the studies by Gulsen et al. [38] and Arshad et al. [31], significantly higher values of the nasal length (Nlength), nasal depths 1 and 2 (Ndepth1 and Ndepth2), and nasal hump (Hump) were reported in men. The convexity of the lower part of the nasal dorsum (Cconv) and nasal bone length (NboneL) were higher in men in the study by Arshad et al. [31], whereas SFC was higher in men in the study by Gulsen et al. [38].

The results reported by Nehra and Sharma [34] indicate a significant correlation between nasal length and upper frontal facial height, inclination of the hard palate vault, and upper facial height. An upturned nose in adults is significantly correlated with maxillary anterior rotation [34]. This report is contrary to the study by Gulsen et al. [38], who found no significant correlation between the nasolabial angle (which is strongly correlated to the upturned nose) and facial skeletal parameters. Moreover, nasal length is correlated with palatal inclination NL (maxillary inclination) [34], similar to findings by Gulsen et al. [38], who observed an association between the nasal base angle and the inclination of the palatal plane.

Meng et al. [39] in their study on young Americans noticed that the proportion between upper and lower nasal height (3 : 1) is stable between the ages of 7-18 and is the same in both sexes. In males, continuous changes in nasal growth were observed between ages 7, 13, and 18. The nose grew more forwards than downwards [1, 39, 42]. Between the ages 13 and 18, the nose moved forwards more by the increasing nasal depth (Prn′-Prn) than by increasing the distance PMV-Prn′. The highest increase in females was noticed from age 7 to age 16. The nose grows more forwards than downwards, similarly as in men. In comparison, Buschang et al. [40] reported that in French-Canadian children, the nasal dorsum growth was upwards and forwards, but also in vertical growth, the nose directed downwards and backwards. These findings are consistent with the study by Robinson et al. [37], who noticed the presence of nasal hump in class II. However, in females, a lower increase in nasal depth was found than in males [11, 39]. Meng et al. [39] concluded that nasal growth in males is still present after the age of 18. However, in females, the nose grows until the age of 16 [34, 39, 41, 42].

Skinazi et al. [35] used Rickett's E-line and the Juanita Line. A sandwich is formed by these two lines and encloses the soft tissue profile in two thirds. Based on these measurements, Skinazi et al. [35] concluded that the mean upper lip, lower lip, chin, and total area were all statistically larger in men. They found no sexual dimorphism in the size of the nose [35]. Referring to nasal dimensions, similar results have been reported by Scavone et al. [8] (Japanese-Brazilian population), and contrary ones—by Anić- Milo**š**ević et al. [7] (investigation in Croatian and American population).

## 5. Conclusions

1. Numerous methods of nasal profile analysis can be found in the literature. These methods describe various numbers of parameters, which have influence on the facial aesthetic
2. The methods by Gulsen et al. as well as by Arshad et al. consider the highest number of variables and thus provide lots of information of potential clinical significance
3. Nasal parameters are correlated to skeletal class and nasolabial angle, positions of upper incisors, and maxillary inclination
4. Nasolabial angle has no sexual dimorphism, irrespective of ethnic group

## Figures and Tables

**Table 1:** Characteristics of included studies.

Author and year of publication	Type of study	Study objective	Ethnic group	Number of subjects	Age range (years)	Test group	Cephalometric characteristics included in analysis	Verification	Results
Gulsen et al. (2006) [38]	RCT	To evaluate the relationship between the facial skeletal and the nasal profile.	Anatolian Turkish adults	262 (167 F, 95 M)	18-30	Subjects with no prior histories of trauma, orthodontic treatment or facial surgery.	27 parameters: 15 facial skeletal, 10 nasal soft tissue, 2 nasal skeletal.	100 randomly selected cephalograms were retracted 2 weeks later by the same orthodontist	The anterioposterior and vertical classifications were found to be significant with analysis of NLA, NMA, and SFC. Sex was significant for nasal length, nasal depths, hump, in addiction to SFC. Facial heights, lengths, the anteroposterior/vertical position of the maxilla and mandible were found to be correlated to nasal length and form.

Arshad et al. (2013) [31]	Analytical study	To determine any significant difference in nasal profiles amongst subjects in sagittal and vertical skeletal patterns, and to determine gender dimorphism if any.	Pakistani origin	119 (81 F, 38 M)	18-40	Subjects with no prior histories of orthodontic treatment, no cranioskeletal syndromes, anomalies and skeletal asymmetries	12 measurements of nasal profile	20 lateral cephalograms were re-evaluated after one week by the principal investigator.	Significant differences between skeletal classes I, II, and III for NLA, NMA, and SFC. Significant differences between males and females for nasal length, nasal depth, columella, convexity, and nasal bone length was found.

Nehra and Sharma (2009) [34]	RCT	To investigate relationship between nasal morphology and vertical maxillary skeletal pattern.	Indian adults	190 (103 F, 87 M)	18-27	Subjects who had undergone orthodontic treatment. Any congenital anomaly or prior history of orthodontic treatment, surgery or trauma to the face.	7 vertical facial skeletal and 6 nasal soft tissue parameters	35 randomly selected lateral radiographs were traced twice by Karan Nehra at an interval of 1 month.	There was a significant correlation between vertical maxillary skeletal and soft tissue parameters. Nasal length significantly correlated with upper anterior facial height and inclination of palatal plane. Upward nasal tip inclination showed a significant negative correlation with inclination of the palatal plane.

Skinazi et al. (1994) [35]	Analytical study	To examine the relative sizes of the component parts of the soft tissue profile in one population of young adults.	French white adults	66 (21 F, 45M)	18-26	Subjects with angle class I occlusion with no history of orthodontic treatment.	Two lines: Rickett's E-line Juanita line	No data	The mean female profile was more convex and the mean male profile was relatively straighter.

Meng et al. (1988) [39]	Analytical study	To determine growth changes in nasal dimensions and morphology relative to the pterygomaxillary vertical (PMV) plane.	Caucasian origin	305 lateral cephalograms (23 F, 17 M)	7-18	No orthodontic treatment performed, class I or end-to-end molar relationship with normal overbite and overjet at the ages of 7 or 8 years; class I molar relationship with normal overbite and overjet at 17 or 18 years of age. A relaxed lip posture. Six or more cephalograms per individual distributed evenly during the age 7 to 18 years.	4 linear variables and 2 angular measurements	54 lateral cephalograms belonging to three male and three female subjects were traced and digitized. After a period of two weeks, these cephalograms were retraced and redigitized.	Increaments in nose height, depth an inclination are complete in girls by 16 years of age, while continuing to increase in males up to and beyond 18 years. In both sexes the ratio of upper to lower nose heights remains approximately 3 : 1. The ratio of the nose depth to sagittal depth: 1 : 2 at 7 years in both sexes 1 : 1,5 (M) and 1 : 1,6 (F) at 18 years Upper nose inclination was similar for both sexes, lower nose inclination was slightly larger in female. Persons with greater increments in nose depth than in nose hight/sagittal depth develop larger upper nose inclinations.
Hwang et al. (2002) [28]	Case- control study	The purpose of this study was to compare the soft tissue profiles obtained from Korean and European-American adults, in order to understand the ethnic differences.	European-American origin	42 (27 F, 15M)	18-34	Subjects with balanced facial esthetics and normal occlusion, no history of orthodontic treatment or extensive restorative dentistry.	10 angular measurements for facial form, 7 linear and angular measurements for lip position	On the basis of an untraced lateral cephalogram, orthodontists (three American orthodontists in European-American origin and three Korean orthodontists in Korean origin) unanimously agreed that each subject in the subsample had a well-balanced face. One investigator traced the lateral cephalogram.	A comparison of the slope of the forehead showed no significant differences between the two groups.
			Korean origin	60 (30 F, 30 M)	17-23	Subjects with normal occlusion, no previous orthodontic treatment or prosthetic replacement of teeth. Subjects who showed class I molar and canine relationship with no or minimal crowding were selected. Cephalograms with the lip at rest.			

Genecov et al. (1990) [36]	RCT	1. To determine the course of nasal development 2. To evaluate the relative position of the nose in relation to the rest of the soft tissue profile during maturation 3. To determine the size, shape and position of the lips during maturation 4. To search for associations between the patterns of hard and soft tissue development.	Caucasian origin	64 (class I—16 F, 16 M) (class II—16 F, 16 M)	7-18	Subjects with no history of orthodontic treatment, allergies or airway problems. Lateral cephalograms were evaluated at three time periods: 1. The mixed dentition 7-9 years; 2. The early permanent dentition 11-13 years; 3. The early adulthood 16-18 years.	25 linear measurements and 4 angular measurements	10 randomly selected tracings were redigitized to evaluate measurement error which was determined to be insignificant.	Anteroposterior growth and subsequent increased anterior projection of the nose continued in both sexes after skeletal growth had subsided. Females had a large proportion of soft tissue development by age 12 while in males continued growth until age 17 resulting greater soft tissue dimensions. During the developmental period, the angular shape and positional relationship of the nose, lips and chin remained relatively constant for both sexes and was relatively independent of the underlying hard tissues.

Robinson et al. (1986) [37]	RCT	To investigate the relationship skeletal facial pattern and soft tissue nasal form.	White American females	123 F	11-20,6	Subjects with no histories of pathology, trauma, surgical intervention, or orthodontic treatment.	3 linear measurements and 2 angular measurements	No data	Patients with straight profiles tended to have straight noses. Convex profiles accompanied convex nasal shapes. Concave profiles were found with concave nasal shapes.

Buschang et al. (1993) [40]	Case- control study	To quantify the childhood and adolescent growth changes of the upper and lower nasal dorsum and to evaluate aspects of nasal structure.	French – Canadian females	111 cephalograms (37 girls)	6-14	A longitudinal sample of 37 girls, each having cephalograms at 6, 10 and 14 years of age.	1 reference line, 3 linear measurements and 4 angular measurements	The cephalograms (made in 6, 10 and 14 years of age) were traced and superimposed by one well-trained cephalometrist. Method errors range between 0.2-0.4 mm.	The upper dorsum rotates upward and forward (counterclockwise) approximately 10^0^ between 6 to 14 years of age. The results clearly indicate that changes in the nasal dorsum are most closely related to angulation changes of the lower dorsum, particularly during adolescence. Rotational changes of the lower dorsum are most closely related with vertical changes at pronasale.

Posen (1967) [41]	Analytical study	To investigate the growth pattern of the nose in normal persons from infancy through adulthood and sexual difference in nasal growth patterns.	Caucasian children	477 cefalograms (15 girls and 15 boys)	0,25-18	Subjects with normal skeletal profile.	6 linear measurements	No data	After the age of 14 years the nose tip did not grow forward to the same extent as did the nasal bones. These resulted in a straightening or humping of the nasal dorsum. Nasal growth changes both size and form were significant after the age of 13 years. The nose tip became more prominent within the total facial profile after 2 to 3 years of age in both groups. Boys had larger nasal component dimensions than girls. Girls had greater degree of maturity in nasal and facial form than did boys in that age.

Magnani et al. (2004) [43]	Case- control study	To assess average values for the nasolabial angle in young Brazilian individuals and assess the occurrence of sexual dimorphism.	Young Brazilian black subjects	36	10-14	Subjects with normal occlusion upon clinical examination and no history of orthodontic treatment.	The nasolabial angle	The caphalometric landmarks were traced by single researcher.	The nasolabial angle was significantly smaller in females.

Fitzgerald et al. (1992) [32]	RCT	To develop a consistent and reproducible method of constructing a nasolabial angle.	Americans	104 (24 F, 80 M)	22-32	Subjects with class I occlusions with good facial balance, no history of orthodontic treatment or facial surgery.	3 nasolabial parameters and 6 skeletal angular measurements	All cephalometric radiographs were retraced at random by the primary examiners and redigitized after a 7-day period.	A reliable method of constructing the nasolabial angle has been devised that includes angulations of the lower border of the nose and the upper lip. There was no statistically significant difference between men and women. No correlation between the soft tissue profile measures and the six skeletal measurements in the well-balanced profile.
Bagwan et al. (2014) [33]	RCT	To evaluate soft tissue parameters for adults, and applying a new method for soft tissue analysis to provide good diagnosis and treatment planning.	Egyptian population	100 (30 F, 70 M)	18-25	Subjects with accepted facial proportions and normal overjet and overbite, Angle's class I occlusion, full complement of permanent teeth, no history of orthodontic treatment or any orthognathic or plastic surgery.	6 lip position parameters, 7 facial form parameters included nasolabial angle	No data	Egyptian population group were found to have more convex faces, protrusive lips and acute nasolabial angles. Males had more convex faces and protrusive lips than females. Egyptian population group had significant deviations from the white standard soft tissue.

Paradowska-Stolarz and Kawala (2015) [29]	Case- control study	To compare the nasolabial angle between the groups of patients with total clefts of the lip, alveolar bone and palate and healthy individuals.	Polish children	118 with cleft (45 F, 73 M)	No data	Subjects with clefts (27 with the bilateral and 91 with the unilateral type of deformity)	The nasolabial angle	No data	In patients with cleft deformities, the nasolabial angle values were smaller than in healthy individuals. Among the patients with clefts, the ones with a bilateral type of deformity are characterized by highest mean values of nasolabial angle.
				101 healthy (69F, 32 M)		The control group was healthy patients with orthodontic treatment needs.			

Kumar et al. (2019) [30]	Cross-sectional study	To evaluate and compare the soft tissue growth changes between males and females of two groups from 8 to 16 years.	No data	160 (group I—40 F, 40 M; group II—40 F, 40 M)	8-16 (group I—8-12 years; group II—12-16 years)	Subjects with skeletal class I, no history of prior orthodontic treatment, no history of bone deformities, or bone diseases, and major illness in the past, no congenital abnormalities affecting growth and development.	8 linear parameters and 4 angular parameters included nasolabial angle	No data	All the parameters increased in their dimension while angle of total facial convexity including nose and nasolabial angle decreases. Among the linear variables, nose height, lip thickness at B point, soft tissue chin thickness and measurements of lips to E-plane were found significant for both subgroups. Males showed a larger value of all the parameters in relation to females.

Aljabaa (2019) [48]	RCT	To establish normal values for the nasal form and its relationship to the other cranial structures among male and female skeletal class I.	Saudi adults	62 (32 F, 30 M)	20-24	Subjects with pleasant facial profile, class I molar and canine relationship, normal overjet and overbite, no crowding, competent lips, no previous orthodontic treatment, no significant medical history, no trauma history, and no craniofacial deformities.	3 nasal size measurements,3 nasal shape angles, 3 angular parameters of the nasal position, 6 linear (horizontal and vertical) parameters	20 cephalometric radiographs were randomly selected and traced twice by the author.	There were statistically significant differences between the Saudi males and females in the nasal length, nasolabial angle, horizontal distance from the nose tip to the incisal edge of the most prominent upper central incisor, and chin. Male had longer dorsa and increased vertical distances from the pronasale to the chin than females. Females had longer vertical distances from the pronasale to the upper lip and larger nasolabial angles than males.

Taha and Ahmed (2020) [49]	RCT	To investigate differences in the nasal profile with different skeletal class groups.	Iraqi adults	90 (class I—15 F, 15M) (class II—15 F, 15 M) (class III—15 F, 15 M)	18-25	Subjects with no previous history of facial trauma, congenital defect, orthodontic treatment or any orthognathic or plastic surgery in the face.	3 nasal size measurements,3 nasal shape angles, 3 angular parameters of the nasal position, 6 linear (horizontal and vertical) parameters, 3 angle determinate the skeletal class	10 randomly selected patients, whose cephalometric radiographs were redigitized and remeasured by the same investigator one month later to help eliminate memory bias.	Significant male-female differences were found in the measurements of nasolabial angle and the horizontal distances relating the nose to the incisal edge of the most prominent maxillary central incisor and to the chin. The angular measurements of the nasal tip projection angle, naso-mental angle and naso-facial angle were also considerable varied among the three skeletal classes.

**Table 2:** Nasal and cephalometric landmarks in the literature (included in the paper nos. 28-30, 32).

Landmarks		Definition
Abbreviation	Name	
G′	Soft tissue Glabella	The most prominent point of the forehead
N′ [28–30]	Soft tissue Nasion	The point of greatest concavity in the midline between the forehead and the nose
N′ [32]	Projected Nasion	The point of intersection of the soft-tissue profile by a line drawn perpendicular to the PMV plane through the nasion
Mn	Midnasale	The halfway point on nasal length (N′- Pr) that divides the dorsum into the upper and lower dorsum
St	Supratip	The point constructed between midnasal and pronasal on the lower third of the nasal dorsum
Pr	Pronasale	The tip of the nose (nasal tip)
Prn	Pronasale	The point constructed with a line drawn parallel to the PMV plane and tangential to the anterior profile of the nose
Prn′	Projected Pronasale	The point of intersection of a line drawn parallel to the PMV plane from the projected nasion (N′) with a line drawn perpendicular to the PMV plane through the pronasale (Prn)
Cm	Columella	The most convex point on the columellar-lobular junction
PCm	Posterior Columella point	The most posterior point of the lower border of the nose at which it begins to turn inferiorly to merge with the philtrum of the upper lip
Sn	Subnasale	The deepest point at which the columella merges with the upper lip in the midsagittal plane
Ls	Labrale superior	The point indicating the mucocutaneous border of the upper lip
Ac	Alar curvature point	The most convex point on the nasal alar curvature
Pg′	Soft-tissue Pogonion	The most anterior point on the chin in the midsagittal plane
N	Nasion	The intersection of the frontal and nasal bones
N1	Nasion 1	The most concave point of the nasal bone
N2	Nasion 2	The most convex point of the nasal bone
R	Rhinion	The most anterior and inferior point on the tip of the nasal bone
S	Sella	Centre of the Sella turcica
ANS	Spina nasalis anterior	The most prominent point of the nasal spine
Ans′	Anterior nasal spine projected to soft tissue	The point of intersection of the soft-tissue profile with a line drawn perpendicular to the PMV plane through the anterior nasal spine (Ans)
Ans^″^	Projected anterior nasal spine	The point of intersection of a line drawn parallel to the PMV plane through projected nasion (N′) with a line drawn perpendicular to the PMV plane through anterior nasal spine (Ans)
PNS	Spina nasalis posterior	The most posterior point of the nasal spine
Go	Gonion	The most posterior and inferior point of the branch of the mandible
Me	Menton	The most inferior bony point of the mandible
Gn	Gnation	The most inferior and anterior point of the mandible
Se	Sphenoethmoid point	The intersection of the shadows of the greater wings of the sphenoid with the floor of the anterior cranial fossa
Ptm	Pterygomaxillary point	The most inferior and posterior point on the pterygomaxillary fissure

**Table 3:** Angular and linear variables (included in the papers of Ref nos. 28-30, 32, 35).

Abbreviation (unit)	Name	Definition	Interpretation
N′-St	The axis of the dorsum	Distance between the soft tissue nasion point and the supratip point	Length of the nasal dorsum
N′-Pr	Nasal length	Distance between the N′ point and the Pr point	Total nasal length
N LTh	Nasal length	Distance between the N′ point and the Pr point	Total nasal length
(1)	Nasal depth (1)	Perpendicular distance between Pr and the N′-Sn line	Sagittal position of the nose tip referring to the face
N Dpt	Nasal depth	Perpendicular distance between Pr and the N′-Sn line	Sagittal position of the nose tip referring to the face
Al.-Pr	Nasal depth (2)	Distance between points Al and Pr	Sagittal position of the nose tip referring to the alar base
Prn′-Prn	Nasal depth	Perpendicular to the PMV plane distance between points Prn′ and Prn on the tip of the nose	The frontal depth of the nose
PMV-Prn′	Sagittal nasal depth	Distance between PMV plane and point Prn′	Sagittal depth of skeleton underlying the pronasale
N′-Prn′	Upper nose height	Distance between points N′ and Prn′	Upper nose height to the point Prn′
Prn′-Ans^″^	Lower nose height	Distance between points Prn′ and Ans^″^	Lower nose height to the point Ans^″^
Hump	Hump	Perpendicular distance between the axis of dorsum and its most prominent soft tissue point	Convexity of nasal dorsum
NBA	Nasal base angle	Angle between the G′-Sn line and the long axis of the nostril	Inclination of the nasal base referring to the face
NMA	Nasomental angle	Angle between the axis of the dorsum and the Pr-WPg line	Relation between nasal dorsum inclination and chin position
SFC	Soft tissue facial convexity	Angle between the lines G′-Sn and Sn-WPg line	Profile convexity
NTP	Nasal tip angle	The angle formed by the axis of the dorsum and PCm tangent	The angle of the tip of the nose
PMV-N′-Prn	Upper nose inclination	The angle formed by the line between PMV plane and point N′ and nasal length (N′-Prn)	The angle of the upper height of the nose
PMV-Ans′-Prn	Lower nose inclination	The angle formed by the line between PMV plane and point Ans′ and Ans′-Prn line	The angle of the lower height of the nose
Dconv	Lower dorsum convexity	Perpendicular distance between the Mn-Pr line and its most prominent point	Convexity of the lower part of the nasal dorsum
Cconv	Columella convexity	Perpendicular distance between the Pr-Sn line and the most anterior point on the convexity of the columella	Convexity of the nasal base
NboneL	Nasal bone length	The line constructed between the N point and the R point	Length of the long axis of the nasal bone
NboneA	Nasal bone angle	The posterior angle between the lines N1-N2 and N2-R	Curvature of the nasal bone
NLA	Nasolabial angle	Angle between the points ctg, Sn, UL	Relationship between the upper lip and the columella
UNLA	Nasal upward tip angle	The posterio-inferior angle formed when the PCm tangent is extended anteriorly to intersect the Frankfurt horizontal plane	The posterio-inferior angle formed by lower border of the nose to the Frankfurt horizontal plane
LNLA	Upper lip inclination	The anterio-inferior angle formed by the PCm-Ls line extended superiorly to intersect the Frankfurt horizontal plane	Inclination of the upper lip to the Frankfurt horizontal plane
GoGn-SN	Inclination of the mandibular plane to the cranium	Inclination the lines S-N to the Go-Gn	The mandibular plane inclination to the anterior cranial fossa
S-Go	Posterior facial height	Distance between points S and Go	Posterior facial height
N-Me	Anterior facial height	Distance between points N and Me	Anterior facial height
N-ANS	Anterior maxillary height	Distance between point N and point ANS	Upper front of the facial height
ANS-Me: LAFH	Lower front of the facial height	Ratio between the middle	Height of the lower facial part
SN-Pp	Inclination of palatal plane	The angle between the Sella-nasion plane and the ANS-PNS line	Inclination of the palatal plane to the anterior cranial fossa
Angle of inclination	Angle of inclination	The angle between the perpendicular drawn from N′ on Se-N′ line and the palatal plane	The angle between entry of the Sella soft tissue nasion and the palatal plane
NF	Nasofacial angle	Formed by the intersection of the soft-tissue glabella to the soft pogonion with the plane of the bridge of the nose	Protrusion of the nose from the facial bones
CL	Columellar to the lip angle (nasolabial angle)	Formed by the intersection of the line from the upper lip to the glabella and the lower border of the nose	The vertical angulation of the nose tip
UN	Upper nose length	Horizontal distance from the nasion to the tip of the nose	Upper length of the nose
ANS	Nose depth	Horizontal distance from ANS to nose tip	Depth of the nose
BN	Lower nose length	Horizontal distance from soft-tissue point A to the tip of the nose	Lower length of the nose

**Table 4:** Characteristics of the studies included—according to Arrive Scale [25].

Authors and year of publication	Study design	Study purpose	Reference standard	Inclusion criteria	Indeterminate results	Exclusion criteria	Spectrum of patients	Analysis method	Analysis criteria	Avoided work-up bias	Avoided diagnostic-review bias	Avoided test-review bias	Intraobserver reliability	Interobserver reliability	Statistical analysis
Gulsen et al. (2006) [38]	1	1	1	1	1	1	0	1	1	1	1	1	1	0	1
Arshad et al. (2013) [31]	1	1	1	1	1	1	0	1	1	1	1	1	1	0	1
Nehra and Sharma (2009) [34]	1	1	1	1	1	0	1	1	1	1	1	1	1	0	1
Skinazi et al. [35]	1	1	1	1	1	0	0	1	1	1	1	1	0	0	1
Meng et al. (1988) [39]	1	1	1	1	1	1	1	1	1	1	1	1	1	1	1
Hwang et al. (2002) [28]	1	1	1	1	1	1	1	1	1	1	1	1	1	1	1
Genecov et al. (1990) [36]	1	1	1	1	1	0	1	1	1	1	1	1	1	0	1
Robinson et al. (1986) [37]	1	1	1	1	1	0	1	1	1	0	0	1	0 (not properly described)	0	1
Buschang et al. (1993) [40]	1	1	1	1	1	1	1	1	1	1	1	1	1	1	1
Posen (1967) [41]	1	1	1	1	1	1	1	1	1	0	0	1	1	1	1
Magnani et al. [28], 2004	1	1	1	1	1	1	0	1	1	1	1	1	1	0	1
Bagwan et al. (2014) [33]	1	1	1	1	1	0	0	1	1	1	1	1	1	0	1
Fitzgerald et al. (1992) [32]	1	1	1	1	1	0	0	1	1	1	1	1	1	1	1
Paradowska-Stolarz and Kawala (2015) [29]	1	1	1	0	1	0	0	1	1	1	1	1	0 (not properly described)	0	1
Kumar et al. (2019) [30]	1	1	1	1	1	0	1	1	1	1	1	1	1	1	1
Aljabaa (2019) [48]	1	1	1	1	1	0	1	1	1	1	1	1	1	1	1
Taha and Ahmed (2020) [49]	1	1	1	1	1	1	0	1	1	1	1	1	1	1	1

**Table 5:** Nasolabial angle in various ethnic groups.

Authors, year of publication	Naso-labial angle (degrees)		Group size		Study group
	Female	Male	Female	Male	
Fitzgerald JP et al. [32], 1992	116.19 ± 9.76	113.55 ± 9.44	24	80	Americans
Hwang HS et al. [28], 2002	109.71 ± 7.60	112.05 ± 9.86	27	15	European-American origin adults
Hwang HS et al. [28], 2002	92.00 ± 9.55	91.11 ± 8.12	30	30	Korean origin adults
Arshad T et al. [31], 2013	98.87 ± 15.76	100.55 ± 14.52	81	38	Pakistani origin
Bagwan AA et al. [33], 2015	96.46 ± 11.30	94.40 ± 10.23	30	70	Egyptian adults
Paradowska-Stolarz AM and Kawala B [29], 2015	112.77 ± 13.17	116.60 ± 11.58	69	32	Poles - control groups
Paradowska- Stolarz AM and Kawala B [29], 2015	101.14 ± 17.51	100.36 ± 18.13	45	73	Poles with cleft lip and palate
Kumar A et al. [30], 2019	103.93 ± 6.78	104.25 ± 6.02	40	40	Indian origin (8-12 years)
	101.05 ± 1.96	101.90 ± 4.67	40	40	Indian origin (12-16 years)
Aljabaa AH [48], 2019	104.19 ± 11.92	96.23 ± 12.74	30	30	Saudi subjects
Taha and Ahmed [49], 2020	101.73 ± 12.15	97.93 ± 9.75	15	15	Iraqi origin, skeletal class I

## Data Availability

All data is a part of the manuscript.

## Abbreviations

NLA: Nasolabial angle
PMV: Vertical pterygomaxillary plane
TVL: True vertical line
Sn: Subnasale point
NMA: Nasomental angle
SFC: Convexity of facial soft tissue
Dconv: Concavity of lower part of nasal dorsum
Ndepth1: Nasal depth 1
Ndepth2: Nasal depth 2
Hump: Nasal dorsum convexity
Prn′-Prn: Nasal depth
TVL-NT: True vertical line-nasal tip
TVL-B: True vertical line-point B
Nlength: Nasal length
Cconv: Convexity of lower part of nasal dorsum
NboneL: Nasal bone length.
